# Supplementary material for: Deciphering Bacterial Community Succession and Pathogen Dynamics in ICU Ventilator Circuits Through Full-Length 16S rRNA Sequencing for Mitigating the Risk of Nosocomial Infections
Source: Microorganisms. 2025 Aug 25;13(9):1982. doi: 10.3390/microorganisms13091982 (PMC12471920; doi:10.3390/microorganisms13091982)
Supplement: Supplementary file 1 [file microorganisms-13-01982-s001.zip › microorganisms-3723508-supplementary.pdf]

Supplementary Information

Table S1. Sample and patient background information.

Day	Sample	Age	Gender	Cause
	VT-7	66	male	Hypercapnic respiratory failure
	VT-9	80	male	STEMI
	VT-11	77	male	IHCA with VF s/p defibrillation x1
Week-1	VT-13	62	female	Acute pulmonary edema with hypercapnia respiratory failure, suspect CAD or asthma attack
	VT-17	34	male	Carbon monoxide poisoning (suicide) with deep coma, metabolic acidosis
	VT-42	71	female	Acute pulmonary edema with hypercapnia respiratory failure
	VT-12	94	female	Right massive pleural effusion with impending hypercapnic respiratory failure
	VT-14	76	female	Acute pulmonary edema and pneumonia with respiratory failure
	VT-23	47	male	In-hospital cardiac arrest
Week-2	VT-71	81	male	Out of hospital cardiac arrest, status post cardiopulmonary circulation resuscitation for 27 minutes then returned of spontaneous circulation, related to hypercapnic respiratory failure
	VT-73	86	female	Hypercapnia with respiratory failure post intubation with ventilator support
	VT-4	81	male	Septic shock, Hypercapnic respiratory failure
	VT-8	51	female	Acute respiratory failure, suspected pneumonia and acute respiratory distress syndrome (ARDS)
Week-3	VT-15	86	female	Hypercapnia with respiratory failure
	VT-18	73	female	Active COVID-19 infection with bilateral pneumonia and respiratory failure
	VT-36	71	male	NSTEMI with acute respiration failure

Table S2. The PCR conditions for the identification of ESKAP pathogens.

Species	Target Gene	Size	Sequence (5′ to 3′)	Reaction Materials Final Volume: 25 l	PCR Condition	Reference
Acinetobacter baumannii	ITS region	208	p-Ab-ITSF: 5′-CATTATCACGG-TAATTAGTG-3′<br>p-AbI-TSB: 5′-AGAGCACTGTG-CACTTAAG-3′	DNA: 100-300 ng<br>Primer: 400 nM<br>Master mix: 5 ul	Pre-denaturation: 94°C 5 min<br>Denaturation: 94°C 30s<br>Annealing: 55°C 30s<br>Extension: 72°C 30s<br>D.A.E. Cycles: 30 cycles<br>Final extension: 72°C 5 min	[1]
Pseudomonas aeruginosa	16S rRNA	956	outer<br>E8F: 5′-AGAGTTT-GATCATGGCTCAG-3′<br>U1510R: 5′-CGGTTACCTTGTTAC-GACTT-3′<br>inner	DNA: 100-300 ng<br>Primer: 400 nM<br>Master mix: 5 ul	outer<br>Pre-denaturation: 95°C 4 min<br>Denaturation: 94°C 30s<br>Annealing: 55°C 30s	[2]

			PA-SS-F: 5′-GGGGGATCTTCGGAC-CTCA-3′<br>PA-SS-R: 5′-TCCTTAGAG-TGCCCACCCG-3′		Extension: 72°C 90s<br>D.A.E. Cycles: 30 cycles<br>Final extension: 72°C 4 min<br>inner<br>Pre-denaturation: 95°C 4 min<br>Denaturation: 94°C 30s<br>Annealing: 58°C 30s<br>Extension: 72°C 60s<br>D.A.E. Cycles: 30 cycles<br>Final extension: 72°C 4 min	
Klebsiella Pneumoniae	16S-23S ITS	130	KB-Pf-F: 5′- ATTTGAAGAGGTT-GCAAACGAT-3′<br>KB-Pr1-R: 5′-TTCAC-TCTGAAGTTTTCTTGTGTTC-3′	DNA: 100-300 ng<br>Primer: 400 nM<br>Master mix: 5 ul	Pre-denaturation: 95°C 15 min<br>Denaturation: 94°C 30s<br>Annealing: 58°C 90s<br>Extension: 72°C 90s<br>D.A.E. Cycles: 35 cycles<br>Final extension: 72°C 10 min	[3]
Staphylococcus aureus	*sodA*	76	sodA F-outer161: 5′-CCAATGTAG-TCAGGGCGTTT-3′<br>sodA R-outer161: 5′-GGTTGGGCTT-GGTTAGTCGT-3′<br>sodA F-inner79: 5′-GCGTGTTCCCATACGTCTAAA-3′<br>sodA R-inner79: 5′-TTGTGAC-TACACCAAACCAAGA-3′	DNA: 100-300 ng<br>Primer: 400 nM<br>Master mix: 5 ul	outer<br>Pre-denaturation: 95°C 4 min<br>Denaturation: 94°C 30s<br>Annealing: 55°C 30s<br>Extension: 72°C 30s<br>D.A.E. Cycles: 30 cycles<br>Final extension: 72°C 4 min<br>inner<br>Pre-denaturation: 95°C 4 min<br>Denaturation: 94°C 30s<br>Annealing: 58°C 30s<br>Extension: 72°C 30s	[4]

					D.A.E. Cycles: 30 cycles<br>Final extension: 72°C 4 min	
Enterococcus faecium	*ddI*	1091	E.faecium-F: 5′-GAG TAA ATC ACT GAA CGA-3′<br>E.faecium-R: 5′-CGC TGA TGG TAT CGA TTC AT-3′	DNA: 100-300 ng<br>Primer: 400 nM<br>Master mix: 5 ul	Pre-denaturation: 95°C 4 min<br>Denaturation: 94°C 30s<br>Annealing: 55°C 30s<br>Extension: 72°C 60s<br>D.A.E. Cycles: 35 cycles<br>Final extension: 72°C 4 min	[5]
Enterococcus faecalis	*ddI*	941	E.faecalis-F: 5′-ATC AAG TAC AGT TAG TCT TTA TTA G-3′<br>E.faecalis-R: 5′-ACG ATT CAA AGC TAA CTG AAT CAG T-3′	DNA: 100-300 ng<br>Primer: 400 nM<br>Master mix: 5 ul	Pre-denaturation: 95°C 4 min<br>Denaturation: 94°C 30s<br>Annealing: 55°C 30s<br>Extension: 72°C 60s<br>D.A.E. Cycles: 35 cycles<br>Final extension: 72°C 4 min	[6]

References

1. Chen, T.-L.; Sin, L.-K.; Wu, R.-C.; Shaio, M.-F.; Huang, L.-Y.; Fung, C.-P.; Lee, C.-M.; Cho, W.-L. Comparison of one-tube multiplex PCR, automated ribotyping and intergenic spacer (ITS) sequencing for rapid identification of Acinetobacter baumannii. *Clinical microbiology and infection* **2007**, *13*, 801-806.
2. Spilker, T.; Coenye, T.; Vandamme, P.; LiPuma, J.J. PCR-based assay for differentiation of Pseudomonas aeruginosa from other Pseudomonas species recovered from cystic fibrosis patients. *Journal of clinical microbiology* **2004**, *42*, 2074-2079.
3. Turton, J.F.; Perry, C.; Elgohari, S.; Hampton, C.V. PCR characterization and typing of Klebsiella pneumoniae using capsular type-specific, variable number tandem repeat and virulence gene targets. *Journal of medical microbiology* **2010**, *59*, 541-547.
4. Banada, P.P.; Chakravorty, S.; Shah, D.; Burday, M.; Mazzella, F.M.; Alland, D. Highly sensitive detection of Staphylococcus aureus directly from patient blood. *PloS one* **2012**, *7*, e31126.
5. Depardieu, F.; Perichon, B.; Courvalin, P. Detection of the van alphabet and identification of enterococci and staphylococci at the species level by multiplex PCR. *Journal of Clinical Microbiology* **2004**, *42*, 5857-5860.
6. Kasimoglu-Dogru, A.; Gencay, Y.E.; Ayaz, N.D. Prevalence and antibiotic resistance profiles of Enterococcus species in chicken at slaughter level; absence of vanA and vanB genes in E. faecalis and E. faecium. *Research in veterinary science* **2010**, *89*, 153-158.
